# A Conductometric Indium Oxide Semiconducting Nanoparticle Enzymatic Biosensor Array

**DOI:** 10.3390/s111009300

**Published:** 2011-09-28

**Authors:** Dongjin Lee, Janet Ondrake, Tianhong Cui

**Affiliations:** 1 Department of Mechanical Engineering, University of Minnesota, 111 Church St. S.E., Minneapolis, MN 55455, USA; E-Mail: djlee@umn.edu; 2 Department of Mechanical Engineering, Ohio Northern University, 525 South Main St., Ada, OH 45810, USA; E-Mail: j-ondrake@onu.edu

**Keywords:** biosensor array, nanoparticle, conductometric sensor, microsensor array, glucose sensor

## Abstract

We report a conductometric nanoparticle biosensor array to address the significant variation of electrical property in nanomaterial biosensors due to the random network nature of nanoparticle thin-film. Indium oxide and silica nanoparticles (SNP) are assembled selectively on the multi-site channel area of the resistors using layer-by-layer self-assembly. To demonstrate enzymatic biosensing capability, glucose oxidase is immobilized on the SNP layer for glucose detection. The packaged sensor chip onto a ceramic pin grid array is tested using syringe pump driven feed and multi-channel I–V measurement system. It is successfully demonstrated that glucose is detected in many different sensing sites within a chip, leading to concentration dependent currents. The sensitivity has been found to be dependent on the channel length of the resistor, 4–12 nA/mM for channel lengths of 5–20 μm, while the apparent Michaelis-Menten constant is 20 mM. By using sensor array, analytical data could be obtained with a single step of sample solution feeding. This work sheds light on the applicability of the developed nanoparticle microsensor array to multi-analyte sensors, novel bioassay platforms, and sensing components in a lab-on-a-chip.

## 1. Introduction

Biosensor technology has evolved synergistically with the advent of nanomaterials possessing novel properties such as mechanical stiffness and strength [1], electrical [2] and thermal conductivity [3], photoemission [4], electromechanical [5] and electrochemical [6] transduction, and catalytic activity [7], *etc*. Nanomaterial-based biosensors [8] have garnered much attention since they can be miniaturized in conjunction with microfabrication techniques to attain low fabrication cost, possible implantation and distributed sensing systems [9]. Leaving such general advantages from the miniaturization, first of all, nanomaterials have size similarity to bioentities such as proteins and DNAs, which facilitates the interactions between transducing nanomaterials and bioreceptors, resulting in high sensitivity and resolution. The incorporation of nanomaterials into devices as a sensing element is beneficial to develop enzymatic biosensors. In general, enzymatic biosensors suffer from the lack of simple immobilization methods and the denaturation of the immobilized enzymes. However, carbon nanotubes and nanoparticles are known to possess a high surface-to-volume ratios, and thereby abundant surface functional groups that can be tethered to biomolecules through a simple self-assembly technique [10]. Moreover, it turned out that enzymes immobilized on nanomaterials retain their activity and structure [11,12]. The immobilization of enzymes onto nanomaterials was demonstrated by the great enhancement of sensor capabilities [13,14].

Most biological processes such as the citric acid cycle are based on the electrostatic interactions and charge transfers with the aid of enzymatic reactions [15]. Consequently, the charges can be readily intercepted by or transferred from/to the nanoscale materials, which is then detected by an external electric circuitry. Therefore, electrochemical sensors are advantageous for biosensing applications using nanomaterials. In addition, nanomaterials provide electrochemical sensors with a delicate path to design new structures and to interface biological recognition events with the electronic signal transduction event [6]. The diverse roles of nanoparticles as: (a) biomolecule immobilization sites; (b) catalyst for electrochemical reactions; (c) electron transfer enhancement; (d) biomolecule labeling; or (e) reactant in electrochemical sensors and biosensors have been reviewed extensively [16]. Most electrochemical sensors were implemented as amperometric or potentiometric type. Both types of sensors require the reference electrode that makes the sensor system bulky. On the other hand, planar type conductometric devices are advantageous in that they can be used for continuous monitoring using the simplicity of the electronic detection and are low-cost due to the possibility of mass production. Furthermore, they can be incorporated into implantable devices for possible *in vivo* applications [17]. In this work, semiconducting nanoparticles were used as an active electrochemical transducing material from which the electrical signal is generated in response to the chemical information in a sample [18]. Particularly, the conductance of nanoparticle thin-films is vulnerable to the charged species, so that a detectable signal can be obtained upon the chemical composition change induced by chemical or biochemical reactions.

Indium oxide (In_2_O_3_) nanoparticles (INPs) were successfully used for pH [19] and neurotransmitter acetylcholine sensing [18] as a type of ion-sensitive field effect transistors (ISFETs). In spite of their excellent sensing properties, nanomaterial thin-films have disadvantages of the variation in electrical conductance from device to device presumably due to the random network nature of the electric path. We addressed this issue by fabricating all-nanoparticle biosensor array that enabled the statistical analysis after a single sample delivery step. It was successfully demonstrated as a glucose biosensor array with the aid of glucose oxidase (GOx) enzyme and a microfluidic sample delivery system, resulting in glucose concentration dependent currents due to the electrochemical properties of the nanoparticles multilayer. Furthermore, statistical analysis was performed in terms of sensitivity and the apparent Michaelis-Mention (MM) constant depending on the channel length of resistors, leading to variable sensitivity and constant apparent MM constants. The sensitivity is found to be dependent on the channel length of the resistor, 4–12 nA/mM for the channel lengths of 5–20 μm, while the apparent MM constant is invariable at 20 mM. This work shed light on the applicability of the developed microsensor array to multi-analyte sensors, novel bioassay platforms, and as a sensing component in lab-on-a-chip systems.

## 2. Experimental Section

### 2.1. Materials

Indium oxide (In_2_O_3_) nanopowders (INP) were purchased from Sigma-Aldrich, and colloidal silica nanoparticles (SNOWTEX^®^-XL) was from Nissan Chemical America Corp. INPs were dispersed into 12 mM HCl (pH 3.9) aqueous solution due to their neutral isoelectric point of 8.7 [20], with the concentration of 50 mg/mL. As-received colloidal silica of 4 g was diluted to 100 mL with deionized water (DIH_2_O) resulting in a concentration of 16 mg/mL with a neutral pH (pH 7.0). Aqueous solutions of polydiallyldimethylammonium chloride (PDDA, Mw = 200–350 k, Sigma-Aldrich) and sodium polystyrene sulfonate (PSS, M_w_ = 70 k, Sigma-Aldrich) were prepared as described previously [21]. The concentration of PDDA and PSS aqueous solution was 1.4 and 0.3 wt%, respectively, with 0.5 M sodium chloride (NaCl). Another set of PSS solution (PSS2) was prepared to maintain a positive surface charge density of INPs inside the PSS aqueous solution during the self-assembly process. The difference of PSS2 from PSS was the pH that was adjusted to 3.9 using HCl [22]. Glucose oxidase (GOx, Sigma-Aldrich, Type VII, lyophilized powder, 50 kU/g, from Aspergillus niger) and standard glucose solutions were formulated in 1× phosphate buffered saline (PBS, pH 7.2, GIBCO, KCl: 2.67 mM, KH_2_PO_4_: 1.47 mM, NaCl: 137.93 mM, Na_2_HPO_4_·7H_2_O: 8.06 mM) as done previously [17]. The concentration of GOx aqueous solution was 1.0 mg/mL with a negative charge at a neutral pH.

### 2.2. Particle Size and Zeta Potential Analysis

A ZetaPlus zeta potential and particle size analyzer (Brookhaven Instruments Co.) was used to perform dynamic light scattering (DLS) for particle sizing and measure zeta potential using phase analysis light scattering (PALS). For DLS, 10 mM of aqueous KNO_3_ was used as a dispersant to make the concentrations of 0.5 and 0.6 mg/mL for INPs and SNPs, respectively, and the signal was collected five times and averaged. Dispersions of INPs and SNPs used for LbL assembly were diluted 100 times for PALS measurement while pH of dilutions was kept at 3.9 and 7.0 for INPs and SNPs, respectively. Ten runs of signal acquisition were done and the electrophoretic mobilities were averaged. All measurement was done at room temperature.

### 2.3. Sensor Chip Design and Fabrication

**Figure 1.**
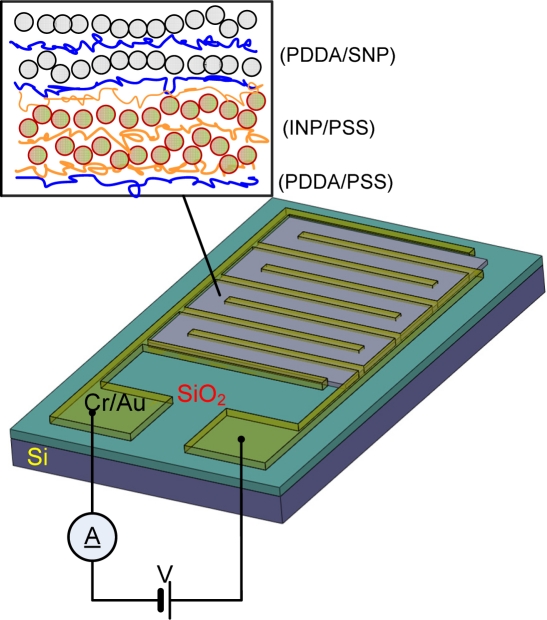
Schematic of device structure: a single sensing sites is shown, where an interdigitated electrode is used for electronic detection.

**Figure 2.**
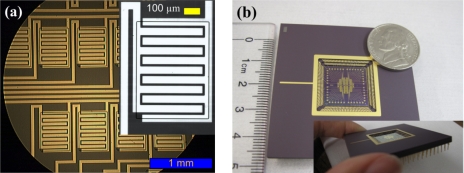

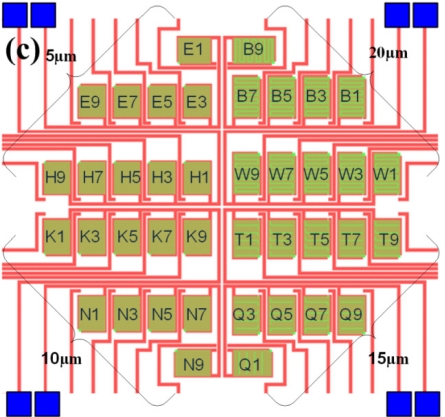
A fabricated sensor array: (**a**) interdigitated electrode patterns with the inset of a single sensing site; (**b**) a chip mounted onto ceramic pin grid array (PGA) by wire-bonding; and (**c**) distribution of sensing sites and connection diagram to XTB system (Rectangles represent common terminal for each letter of sensing sites).

Chromium (Cr, 300 Å) and gold (Au, 1,000 Å) were electron-beam evaporated on a 4 inch silicon wafer with thermally grown oxide 2 μm thick. Photolithography was used to fabricate interdigitated metal electrodes as shown in Figure 1 that demonstrates a single sensing site. 16 sensor chips with a size of 15.5 mm × 15.5 mm were embedded on a 4 inch silicon wafer. Each chip contained 40 sensing sites that were accommodated within a circular microchamber with a diameter of 8 mm. The channel gaps of 5, 10, 15, and 20 μm between two interdigitated electrodes were designed in a single chip to evaluate the effect on device performance. The number and length of fingers in a single sensing site are 5 and 400 µm, respectively, as shown in Figure 2(a). The 2nd lithography was used to fabricate the opening window of photoresist to assemble nanoparticles as a sensing element only on the channel area as depicted in Figure 2(a) inset. The silicon wafer was treated with oxygen (O_2_) plasma at a power of 100 W for 1 min with O_2_ flow rate of 100 sccm (standard cubic centimeter) to make the surface hydrophilic for the subsequent aqueous layer-by-layer (LbL) assembly of polyelectrolytes and nanoparticles.

### 2.4. Layer-by-Layer Assembly of Nanoparticles

The LbL assembly of nanoparticles was conducted on the wafer scale under atmospheric pressure and room temperature. The precursor layer of (PDDA/PSS)_2_ was assembled for surface charge enhancement. INPs were assembled as a semiconducting channel material alternately with the negatively charged PSS2. After assembly of five bi-layers of (INP/PSS2), SNPs were deposited with a pair of the positively charged PDDA as an enzyme immobilization site due to the abundant charged surface groups [23]. The dipping time for INPs and SNPs was 14 and 4 min, respectively. The assembling time for pairing polyelectrolytes (PDDA, PSS, and PSS2) was 10 min. The final thin-film structure was [(PDDA/PSS)_2_ (INP/PSS2)_5_ (PDDA/SNP)_6_]. The lift-off was conducted in the acetone under ultra-sonication to leave nanoparticle thin-film only on the channel, followed by dicing into individual chips. The additional layer of (PDDA/GOx) was assembled as a bioreceptor as reported previously [17] to promote the oxidation of glucose.

### 2.5. Sensor Package

LbL assembled chips were mounted onto a 256 lead ceramic pin grid array (PGA, Global Chip Materials LLC) and all electrodes in 40 sensing sites were wire-bonded as shown in Figure 2(b). The distribution of sensing sites and connection diagram into XTB systems (TSI Inc.) are shown in Figure 2(c). The sensing sites are grouped according to the channel length (5 μm: E and H; 10 μm: K and N; 15 μm: Q and T; 20 μm: W and B) to apply the bias voltage readily. The source electrodes in each group of sensing sites were connected to common terminals that were expressed in blue rectangles in Figure 2(c). The drain electrodes were connected to the electrometers in XTB system.

### 2.6. Sensor Test

**Figure 3.**
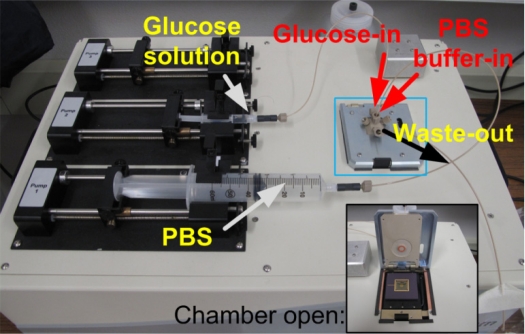
A photograph of multichannel sensing system equipped with the syringe pump driven sample delivery: Inset shows the microchamber at open position.

All sensor array tests were performed in XTB system shown in Figure 3. Standard glucose solutions and washing phosphate buffered saline (PBS) were delivered automatically by syringe pumps at a rate of 100 and 500 μL/min, respectively. A multichannel electrochemical characterization was performed in the following way. Firstly, the microchamber was filled with a washing buffer and incubated for 5 min. The target glucose solutions were injected sequentially with the intermediate rinsing with PBS buffer. One concentration of glucose was fed for 1 min to ensure that the target glucose solution filled microchamber. It was incubated for 1 min for enzymatic reaction to occur and I–V measurement was performed on the range of −1 to 1 V with a voltage step of 5 mV for 40 s. At the same time the current was acquired with a sampling frequency of 10 Hz. Next, the washing buffer was fed to rinse the sensor surface continuously for 2 min, and a new glucose solution was delivered, followed by I–V measurements as described above.

## 3. Results and Discussion

**Figure 4.**
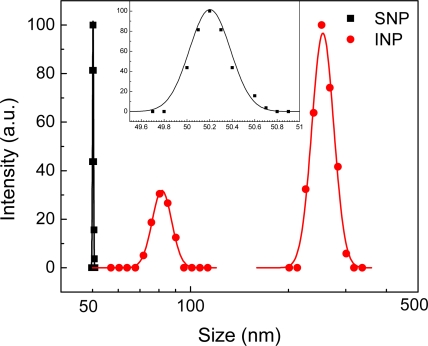
Particle size distribution using dynamic light scattering (DLS): INP has two broad peaks at 81.7 nm and 255.1 nm, while SNP distribution reveals one sharp peak at 50.2 nm with polydispersity of 0.005.

To study the possibility of LbL self-assembly of nanoparticles and estimate the film hierarchy, zeta potential and size measurements were performed. For electrostatic LbL assembly to happen the nanoparticle dispersions should be stable and have enough surface charge. Furthermore, semiconducting nanoparticles must form the electrical contact each other to develop the percolation path through the nanoparticle film [24]. In order to determine the zeta potential (*ς*) of nanoparticles used, the Smoluchowski equation was used based on the electrophoresis, which requires the knowledge of the dielectric constant and the viscosity of the surrounding medium as follows:

(1) $$\mu_{\mathrm{ep}}=\frac{{ɛ}_{r}{ɛ}_{0}\varsigma }{\eta }$$

where *μ* is the electrophoretic mobility of particles, *ɛ_r_* the dielectric constant of the dispersing medium, *ɛ*_0_ the permittivity of the free space, *ς* the zeta potential, and *η* the dynamic viscosity of the medium. The mobility of INPs and SNPs was found as 1.8 ± 0.1 and −3.9 ± 0.2 (×10^−11^ m^2^ V^−1^s^−1^), respectively, from which zeta potentials were calculated as 22.53 ± 0.8 and −48.1 ± 1.2 mV. Therefore, the formulated nanoparticle dispersions had enough charge for LbL assembly. The DLS data were fitted to a lognormal particle size distribution, which has the probability density function as follows [25]:

(2) $$f\left(d\right)=\frac{1}{\sqrt{2\pi }\mathrm{dln}\sigma }\;\exp\;\left[-\frac{\left(\ln\;d-\ln\;d_{m}\right)}{2\ln^{2}\sigma }\right]$$

where *d_m_* is the mean diameter and *σ* is the standard deviation of the distribution. The lognormal distributions of SNPs and INPs are shown in Figure 4. SNPs are monodispersed with the mean diameter of 50.2 nm and the polydispersity of 0.005 as shown in the inset, while INPs show two broad peaks at 81.7 nm and 255.1 nm. INPs are polydispersed due to the possible agglomeration caused by the relatively weak surface charge.

**Figure 5.**
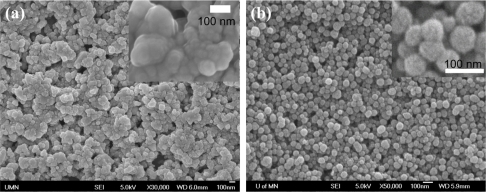
SEM images of INP- and SNP-terminated surface: (**a**) (PDDA/PSS)_2_ (INP/PSS2)_5_; and (**b**) (PDDA/PSS)_2_ (INP/PSS2)_5_ (PDDA/SNP)_6_.

The surface morphology of INP- and SNP-terminated surfaces, (PDDA/PSS)_2_ (INP/PSS2)_5_ and (PDDA/PSS)_2_ (INP/PSS2)_5_ (PDDA/SNP)_6_, were characterized with scanning electron microscopy (SEM, Jeol 6500). A 50 Å of platinum was sputter-coated and SEM images were obtained at an acceleration voltage of 5 kV. SEM images of INPs and SNPs layer are shown in Figure 5, where enlarged images are embedded in the inset to substantiate the particle sizes and contacts among particles. The INP layer is composed of individual nanoparticles and agglomerates whereas the SNP layer has the uniform individual particles presumably due to their own zeta potentials. The SEM of INPs validates the existence of big agglomerations which were found as the mean diameter of 255.1 nm in DSL measurement. However, INPs were populated enough to form electrically conductive channels in the thin-film.

**Figure 6.**
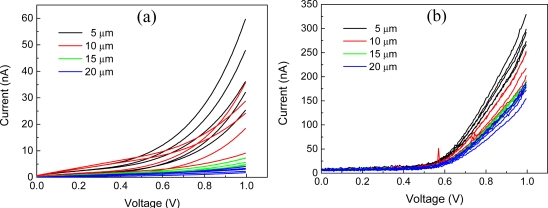
I–V characteristics of nanoparticle resisters: (**a**) in the ambient air; and (**b**) in PBS buffers; multiple curves for each channel length come from 5 sensing sites.

I–V characteristics of nanoparticle resistors on the range from 0 to 1 V in ambient air and PBS are shown in Figure 6. Multiple curves for each channel length came from representative five sensing sites. I–V curves in the ambient air, as shown in Figure 6(a), indicate that the nanoparticle multilayer forms Schottky contacts between nanoparticles [24] and/or nanoparticle multilayer and metal electrodes. This is different from LbL assembled carbon nanotube (CNT) resister that showed the ohmic contacts [18,20]. Particularly, for the sites with 5 and 10 μm channel gap the bias voltage greater than 0.6 V is large enough to overcome the interparticle barriers [24] to allow the significant current, while it is still low for the devices with channel gaps of 15 and 20 μm. On the other hand, currents at PBS, as shown Figure 6(b), are about 30 times higher than those at atmosphere. It is also entirely different from the LbL assembled CNT resistor [21], where the conductivity abruptly drops when an aqueous sample solution was added presumably due to the swelling of multilayer film resulting in reduction of interconnection among CNTs. In addition, it is noticeable that I–V curves showed diode-like behavior with a consistent transconductance to the channel length on the bias voltage larger than 0.6 V that was necessary to overcome the interparticle barrier. Once the barrier was vanquished the current behavior looked like the resistor.

**Figure 7.**
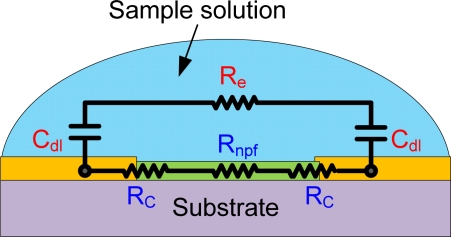
An equivalent electric circuit of nanoparticle resistor in a sample solution: Miniaturized sensor array and the application of DC voltage give rise to *C_dl_* drastically so that analyte dependent currents are obtained through nanoparticle thin-film rather than bulk sample solution.

An equivalent electrical circuit of nanoparticle sensors on the region where the transconductance was observed is illustrated in Figure 7. The intrinsic resistance in electrolyte solution (*R_e_*) and the electric double layer capacitance (*C_dl_*) of metal electrodes are in series. In addition, they are parallel to the contact resistances (*R_c_*) and nanoparticle film resistance (*R_npf_*) including interparticle barrier. Raguse *et al.* [26] demonstrated that nanoparticle chemoresistors sensed the analytes in ionically conductive aqueous solutions by controlling the ratio of *R_npf_* to the impedance (*R_e_* and *C_dl_*) through the electrolyte. We observed much lower current without nanoparticle film than with the the film in the channel gaps tested. Due to the great amount of *C_dl_* in the miniaturized chemoresistors and use of direct current (DC) in I–V measurement, the combinative impedance through bulk solution is assumed to be huge and constant, whereas *R_c_* and *R_npf_* are very sensitive to their microenvironment as discussed I–V curves in PBS. Therefore, the electronic conduction corresponding to analytes concentration occurs preferentially through the nanoparticle thin-film rather than bulk sample solution. The monitoring of *R_npf_* is the key role in nanomaterial sensors because of their high surface-to-volume ratio and ability to make tremendous contacts. Specifically, the sensing current greatly increased in biosensors with the enzymatic layer, which produced new charged ions continuously under the assumption of enough substrates.

**Figure 8.**
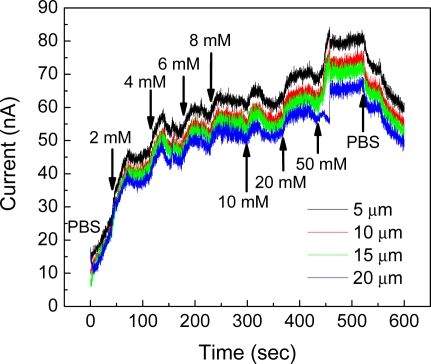
Time response curves from one sensing site per channel length at bias voltage of 0.7 V: the sample was fed constantly without intermediate washing.

Time responses of each channel length to increasing glucose concentrations at the bias voltage of 0.7 V are shown in Figure 8. Unlike the aforementioned testing scheme, one glucose concentration was fed constantly with a rate of 100 μL/min for a period of time and another concentration of glucose was injected without intermediate washing during the application of bias voltage. Even though output currents were sometimes unstable, presumably due to the fluidic instability and mixing in the microchamber caused by continuous delivery of samples while the acquisition of the current, increasing output currents were observed with increasing glucose concentration. The products in GOx enzymatic reaction, especially hydrogen ions, play an important role in modulating *R_npf_* through the protonation of surface hydroxyl groups. Hydrogen ions are generated through either the hydrolysis of gluconic acid or electrocatalytic oxidation of hydrogen peroxide. The concentration of hydrogen ions produced is maintained constant locally in the vicinity of GOx from which ions diffuse into the nanoparticle thin-film or disappear toward the bulk solution due to the buffering power of PBS. The hydrogen ion in the proximity of nanoparticle results in protonation/deprotonation of surface functional groups, thereby the conductance change of nanoparticle thin-film. More positive surface charge develops more conductive channel inside n-type INPs [19]. The effect of other ions other than hydrogen ion on the performance was small compared to the one of hydrogen ions.

**Figure 9.**
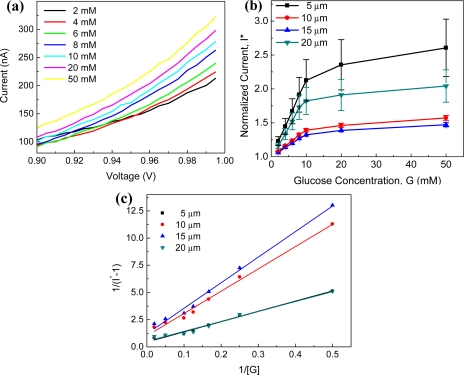
Glucose sensing results of nanoparticle resistor array: (**a**) I–V curves on the range from 0.9 to 1.0 V at various glucose concentrations extracted from one 10 μm channel device; (**b**) normalized currents with respect to the current in PBS at the bias voltage of 1.0 V *versus* glucose concentration; and (**c**) Lineweaver-Burke plot of normalized current, I^*^ and glucose concentration, [G]: Error bas in (b) indicate standard error.

Even though discernible I–V curves were not found at the low bias voltage that is not enough to overcome interparticle barriers, I–V curves on the range from 0.9 to 1.0 V at various concentration of glucose are consistent as illustrated in Figure 9(a) from one 10 μm channel sensing site. The concentration dependent currents are observed at higher bias voltage. The currents (*I*) at the bias voltage of 1.0 V at various glucose concentrations were extracted and normalized with the one in PBS. Normalized currents (I^*^) with standard error as an error bar are shown in Figure 9(b) as a function of glucose concentration at different channel lengths. Although the current level is different from site to site within the same channel length, saturation effects are observed in normalized currents (I^*^) due to the enzyme kinetics. Furthermore, decreasing normalized currents are observed with increasing channel length, while devices with 20 μm channel gap showed higher current than the ones with 5 and 10 μm channel gap. It is supposed that the resistance of the nanoparticle film (*R_npf_*) becomes larger than the contact resistance (*R_c_*) in Figure 7, making it possible to lower the current in PBS leading to higher normalized currents from 20 μm than the ones from other channel gaps. To evaluate enzyme kinetics, the Lineweaver-Berk plot was constructed as shown in Figure 9(c), demonstrating the average $K_{m}^{\text{app}}$ value of about 20 mM for the sensing sites with 5, 10, and 15 μm and 17.7 mM for the ones with 20 μm.

The testing results are summarized in Table 1. The sensitivity found was different, depending on the channel length, decreasing sensitivity being observed with increasing channel length. On the other hand, constant apparent MM constants at about 20 mM were observed, which was thought to be characteristic of immobilized GOx enzymes on silica nanoparticles. The difference in the number of sensing sites considered originates from the failure of wire-bonding. It is interesting to see different behaviors in the sites with 20 μm channel length in the form of an increased sensitivity and reduced apparent MM constant. It seems that charge carriers are transferred through bulk solution due to decreased *C_dl_*, where the advantages of microsensors may disappear. The increased current through the bulk sample solution played a role of higher affinity of GOx to the glucose. The extracted MM constants are smaller than the free GOx enzyme but larger than LbL assembled GOx on CNTs. For comparison, MM constant of GOx immobilized on CNTs through LbL assembly showed 14.2 mM [27]. It is noticeable that MM constant (the affinity) is dependent on the immobilization site presumably due to the steric effect. Furthermore, it is noted that the sensing area plays an important role in sensitivity on the range of physiological glucose level, suggesting the optimization of electrode design. It seemed that the sensitivity increased with decrease in channel gap under the constant activity of glucose oxidase ( $K_{m}^{\text{app}}$), since the resistance of nanoparticle film (*R_npf_*) increased with the channel gap. On the other hand, the breakdown of glucose was seemingly catalyzed at the enhanced enzymatic activity in 20 μm channel gap, resulting in higher sensitivity than in 10 and 15 μm channel gaps.

## 4. Conclusions

An enzymatic nanoparticle biosensor array has been fabricated using INPs and SNPs and successfully demonstrated as an ion-sensitive conductometric glucose biosensor. The nanoparticle resistor showed different I–V characteristics in ambient air and an aqueous PBS environment, presumably due to the charge carrier transfer mechanism. Glucose was successfully detected in most sensing sites within a chip with a single sample delivery shot. The current dependent on glucose concentration was normalized, averaged, and analyzed, and the performance of sensor array was evaluated statistically in terms of sensitivity and apparent MM constant. The sensor array showed a linear response over the physiological range of glucose, yielding a sensitivity of 4–12 nA/mM and MM constant of 20 mM. The developed nanoparticle conductometric sensor array has potential applications in multiplexed sensors [28], various bioassay platforms and as the sensing part in the lab-on-a-chip.

## Figures and Tables

**Table 1.** Summary of glucose biosensor array testing.

**Channel length (μm)**	**# of devices considered**	**Sensitivity (nA/mM)**		**Apparent Michaelis-Menten Constant (**$K_{m}^{\mathrm{app}}$**, mM)**
		**Mean**	**Standard deviation**	
5	10	11.9	3.9	20.3
10	8	5.7	2.6	20.1
15	7	4.4	1.5	19.6
20	7	6.9	2.9	17.7
